# Spatiotemporal heterogeneity in meteorological and hydrological drought patterns and propagations influenced by climatic variability, LULC change, and human regulations

**DOI:** 10.1038/s41598-024-56526-z

**Published:** 2024-03-12

**Authors:** Yunyun Li, Yi Huang, Yanchun Li, Hongxue Zhang, Jingjing Fan, Qian Deng, Xuemei Wang

**Affiliations:** 1 Ecological Security and Protection Key Laboratory of Sichuan Province, Mianyang Normal University, Mianyang, 621000 China; 2 School of Water Conservancy and Civil Engineering, Northeast Agricultural University, Harbin, 150030 China; 3 College of Water Resources and Hydropower, Hebei University of Engineering, Handan, 056038 China

**Keywords:** Meteorological drought, Hydrological drought, Drought evolution, Drought propagation, Influencing mechanism, Climate-change impacts, Environmental social sciences, Hydrology

## Abstract

This study aims to quantify meteorological–hydrological drought propagations and examine the potential impacts by climatic variability, LULC change (LULC), and human regulations. An integrated observation-modeling framework quantifies drought propagation intervals and assesses mechanisms influencing hydrological droughts. Meteorological droughts are characterized using the Standardized Precipitation Evapotranspiration Index (SPEI), and hydrological droughts are assessed through the Standardized Streamflow Index (SSI) across diverse climatic zones. Cross-correlation analysis between SPEI and SSI time series identifies the lag time associated with the highest correlation as the drought propagation interval. Mechanisms are investigated via a coupled empirical-process modeling framework incorporating the Soil and Water Assessment Tool (SWAT). Discrepancies between simulated and observed SSI time series help quantify the extent of human regulation impacts on hydrological drought characteristics and propagation. The Yellow River Basin (YRB), divided into six subzones based on climate characteristics, is selected as the case study. Key findings include: (1) Meteorological droughts were extremely severe across most YRB during the 1990s, while the 2000s showed some mitigation primarily due to precipitation increases. (2) Hydrological droughts and propagation times from meteorology to hydrology demonstrated substantial spatiotemporal variability. In general, summer propagation times were shorter than other seasons. (3) Propagation times were shorter in arid regions with cropland or built-up land cover versus grassland and woodland, while the reverse held for humid regions. (4) Human regulations prolonged propagation times, likely due to reservoir regulations designed to overcome water deficits. While the YRB is the focus of this paper, the methodologies and findings are applicable to other regions worldwide to enhance drought forecasting and water resource management. In various hydrological and climatic contexts worldwide.

## Introduction

Drought is a complex natural phenomenon that occurs when water availability across different components of the hydrological cycle falls below expected levels^1–4^. Droughts, which stem from hydrological deficits, can be classified into four main types: meteorological, hydrological, agricultural, and socioeconomic^5,6^. In the past few decades, these phenomena have had profound impacts on societies around the globe. Meteorological and hydrological droughts, in particular, have led to significant disruptions in economic activities, ecological balance, and the functionality of public water systems^7,8^. Specifically, meteorological drought is defined by a prolonged period of below-average precipitation, leading to a shortfall in surface and subsurface water supplies. Hydrological drought, on the other hand, refers to a situation where there is a sustained period of reduced water content in streams, rivers, lakes, and reservoirs, often as a consequence of meteorological drought. While both types of drought are linked through the hydrological cycle, the distinction lies in their primary indicators: meteorological drought is indicated by reduced precipitation, whereas hydrological drought is indicated by diminished water bodies and streamflows. In the past few decades, these phenomena have had profound impacts on societies globally. Meteorological and hydrological droughts have caused significant disruptions in economic activities, ecological balance, and the functionality of public water systems. This study, therefore, focuses on these two drought types. Drought typically begins with negative hydro-climatic signals that propagate through interconnected hydrological subsystems, including soil systems, surface water bodies, and groundwater, extending from water storage in the landscape to vegetation stress and human water demand. Meteorological drought is a critical driver for other drought types, with persistent or high-frequency meteorological drought often leading to hydrological and other types of drought^9,10^. As meteorological water deficits propagate through terrestrial systems, it takes time for reduced precipitation and moisture supply to manifest as deficiencies in rivers, lakes, reservoirs, and groundwater. Hence, studying the propagation time and influencing mechanisms from meteorological to hydrological drought provides important basic support for formulating drought early warning programs and preventive measures.

Drought propagation, the process by which a lack of precipitation leads to a sequence of drought conditions across different environmental domains, is influenced by a complex interplay of factors. These include climate variability, alterations in land use and cover, infrastructure developments like reservoirs, water extraction, and broader human interventions. Current studies mainly focus on assessing the comprehensive impacts of climate change and human activities on drought propagation^11–13^. For example, studies have shown that large-scale atmospheric patterns such as ENSO and AO can extend the onset of hydrological drought following meteorological drought^9,14^. Additionally, the transition from meteorological to agricultural drought is exacerbated by rising temperatures and reduced soil moisture, although irrigation practices may mitigate some impacts^15^. In semi-arid regions like the Heihe River basin, climate change appears to prolong drought onset, while human activities can either shorten or disrupt this progression^15,16^.

The interplay between land use/land cover (LULC) change, human regulations, and drought propagation in diverse climatic regions has not been sufficiently explored, particularly in terms of the mechanistic impacts. To effectively mitigate and prepare for droughts, it is crucial to distinguish between the direct effects of LULC changes and the indirect effects of human regulations. For example, alterations in watershed landscapes and LULC characteristics significantly influence hydrological processes such as rainfall infiltration, runoff generation, and groundwater recharge/discharge. These changes, driven by human activities, can indirectly modify regional hydrological cycles and affect the timing of drought propagation^17,18^.

Moreover, human regulations, including the development and utilization of water resources like storage infrastructure and irrigation systems, play a dual role in the hydrological drought progression^19,20^. On one hand, they can exacerbate the effects of prolonged meteorological droughts by disrupting the natural flow of river networks and altering retention times^21,22^. On the other hand, practices such as irrigation can increase soil moisture to alleviate agricultural drought, yet they may also deplete surface water levels and hasten the onset of hydrological drought^23–26^. These anthropogenic factors thus impart significant, albeit contrasting, influences on drought dynamics, especially in intensively managed basins such as those in China.

To enhance the management and adaptation of the coupled human-water system under increasingly severe drought conditions, an integrated understanding of how LULC change and human regulations interact to influence the spatiotemporal heterogeneity of drought propagation is imperative. Investigating the nexus of climate change, LULC change, and human regulations is essential for improving our ability to predict drought progression and manage water resources more effectively. This holistic approach will provide a more nuanced understanding of the transition from meteorological to hydrological drought, informing strategies that can better balance the demands of human activities with the sustainability of water resources.

Current methods for studying drought propagation fall into two categories: statistical analysis and hydrological modeling^11^. Hydrological models explore physical mechanisms governing propagation, while statistical techniques like correlation analysis and machine learning identify climatic and watershed controls. Here, we apply Pearson correlation coefficient analysis to quantify meteorological-to-hydrological drought propagation times.

Additionally, assessing climate change and human impacts on hydrological processes requires comparing natural and managed catchments. Common comparative approaches include: (1) large-scale screening approach^27^; (2) paired catchments approach^28^; (3) upstream–downstream approach^29^; (4) pre-post disturbance approach^30^; (5) tributary-comparison approach^31^; and (6) observation-modeling approach^32^. The first two approaches require extensive hydrological records across many catchments. The third and fourth approaches run into nonlinearities between monitoring stations or convoluted anthropogenic–climatic interactions. The fifth approach lacks socioeconomic indicators for reference tributary selection. In contrast, the sixth approach enables quantitative attribution of meteorological–hydrological connections and underlying natural and anthropogenic mechanisms^33^. Therefore, this study utilizes an observation-modeling framework to quantify how climatic, land use/land cover, and human regulation changes influence drought characteristics and propagation in the YRB, using simulated and observed hydrological data.

The Yellow River Basin (YRB) is a critical water source for over 140 million people and 15% of China's cropland, yet has a long history of severe and intensifying droughts^34,35^. Recent decades show rising drought frequency and duration, with the most extreme event in 1997 resulting in 700 km of dried river channel for 226 days. Despite extensive development of over 30 large reservoirs to regulate flows, drought continues to disrupt ecological, agricultural, and socioeconomic systems^36,37^. While numerous studies have examined drought issues in the YRB, including multivariate drought index development, drought frequency analysis, and drought risk assessment^38–43^, knowledge gaps remain exploring the mechanistic drivers of climatic variation, LULC change, and human regulation on drought evolution and propagation. As an intensely managed basin with inherent aridity and hydro-climate diversity, the YRB provides an ideal case study for investigating how meteorological drought signals propagate into hydrological drought events. By revealing the processes governing drought development and propagation in this complex human-natural system, improved hydrological drought monitoring and early warning systems can be developed by Water Resources Management Center and Drought Prevention and Disaster Reduction Department.

The primary objectives of this study are three-fold: (1) Investigate multi-scale temporal and spatial evolution dynamics of meteorological and hydrological droughts; (2) Quantify propagation times from meteorological to hydrological drought; and (3) Reveal potential influence mechanisms of climatic factors, land use/land cover (LULC) changes, and human regulations on hydrological drought and propagations.

## Methodologies

### Meteorological drought index

Selecting or developing appropriate drought indices is crucial for investigating complex drought evolution dynamics, propagations, and governing mechanisms. Numerous drought indices have been proposed, including meteorological indices such as the Standardized Precipitation Index^44^ (SPI), Precipitation Anomaly Percentage^45^ (PAP), Standardized Precipitation Evapotranspiration Index^46^ (SPEI), and Reconnaissance Drought Index^47^ (RDI). The SPEI and RDI are advantageous for capturing meteorological drought as they incorporate precipitation and temperature as key driving factors^48,49^. Compared to the RDI, the SPEI is more sensitive to environmental fluctuations with clearer evaluation criteria, assessing deviations in precipitation minus evapotranspiration from normal conditions^50^.

Therefore, this study utilizes the SPEI as the meteorological drought index. SPEI fits a distribution function to precipitation-evaporation differences, enabling sensitivity to environmental changes and effective correlations with hydrological drought. Details on SPEI calculation and classification are available in Shang et al.^50^ and Table 1.

**Table 1:** The classification of SPEI and SSI.

Drought grade	SPEI	SSI
No drought	$SPEI > - 0.5$	$SSI > - 0.5$
Abnormally dry	$- 1.0 < SPEI \le - 0.5$	$- 1.0 < SSI \le - 0.5$
Moderate drought	$- 1.5 < SPEI \le - 1.0$	$- 1.5 < SSI \le - 1.0$
Severe drought	$- 2.0 < SPEI \le - 1.5$	$- 2.0 < SSI \le - 1.5$
Extreme drought	$SPEI \le - 2.0$	$SSI \le - 2.0$

The water balance equation, based on the principle of conservation of mass, provides reasonably reliable estimates of actual evapotranspiration (AET) at the catchment scale by calculating AET as the residual of precipitation and runoff terms. However, it is limited by the availability and accuracy of the other water budget components, and is sensitive to errors in precipitation and streamflow measurements. To overcome these limitations in quantifying the atmospheric moisture deficit, potential evapotranspiration (PET) is utilized to derive the Standardized Precipitation Evapotranspiration Index (SPEI) for drought analysis.

There are three primary methods for estimating PET—the Penman-Monteith^51^, Priestley–Taylor^52^, and Hargreaves equations^53^. The Penman–Monteith approach calculates PET based on aerodynamic and radiative factors, requiring extensive meteorological data inputs of wind speed, humidity, radiation, and temperature. In contrast, the Priestly–Taylor method utilizes radiation and temperature to determine PET responses to climate variability. The Hargreaves equation only requires temperature and extraterrestrial radiation, providing reliable PET estimates in arid regions but performing poorly in humid environments or with significant wind. Critically, Hargreaves does not account for moisture limitation effects on PET. Among the available options, the Penman–Monteith equation has the most rigorous theoretical basis and fully considers the multiple factors influencing evapotranspiration like atmospheric flow and environmental interactions, providing robust PET estimations across regions and climate conditions. Therefore, this study adopts the Physically-based Penman–Monteith method to calculate PET for deriving SPEI drought indices.

### Hydrological drought index

Appropriate meteorological and hydrological drought indices are foundational for achieving the study objectives. For characterizing hydrological drought, several standardized indices have been developed including the Surface Water Supply Index^54^ (SWSI), Palmer Hydrological Drought Index^55^ (PHDI), Standardized Runoff Index^56^ (SRI), and Standardized Streamflow Index^57^ (SSI). Among these, the SSI is advantageous due to simpler calculation and lower data requirements^7^. The SSI effectively depicts water storage dynamics in rivers and lakes, enabling assessment of climate and human impacts. The streamflow simulated by the swat model in this study is only affected by climate and land use changes, while the observed streamflow is influenced by climate change and human activities (including land use changes and human regulation) Therefore, simulated streamflow-based SSI is utilized to characterize hydrological droughts under climate and LULC change scenarios, while observed streamflow-based SSI represents hydrological droughts under coupled climate change and human activity scenarios. Details on SSI computation are available in Svensson et al.^57^, with drought classification matching that of the SPEI (Table 1).

### SWAT model

Appropriate model selection and performance assessment provides confidence in subsequent investigations of complex drought dynamics. The Soil and Water Assessment Tool (SWAT) is widely applied for simulating complex hydrological processes under climate variation and land use/land cover (LULC) change scenarios^58–62^. Hence, the SWAT model is selected to simulate the hydrological process in this study.

Because the SWAT hydrological model requires 2–3 years of flow data to preheat the model in the early stages of operation, eliminating the default value of model parameters as zero. Therefore, the research period in this study is from 1968 to 2010. To conduct research in different time periods, the change point analysis with Mann-Kendell trend test on the naturalized streamflow time series from 1968 to 2010 at five hydrological stations. The results showed that there was one change point in the naturalized streamflow time series that occurred in 1990. Hence, based on the change point in 1990, the study period (1968–2010) is divided into baseline period (1968–1990) and impact period (1991–2010). The impact period is further divided into two periods of 1991–2000 (1990s) and 2001–2010 (2000s) according to intergenerational differences.

To accurately represent variable underlying surfaces, this study implements the time-varying parameter approach in SWAT proposed by Li et al.^17,63^ calibrating the model for three periods (1968–1990, 1990s, 2000s) using corresponding LULC maps. This enables aggregated characterization of distinct LULC impacts on drought propagation.

SWAT model performance was evaluated using the Nash–Sutcliffe efficiency (NSE), coefficient of determination (R2), and percent bias (PBIAS) metrics^64,65^. According to Moriasi et al^66^, monthly simulations are considered satisfactory if NSE > 0.5, R^2^ > 0.6, and |PBIAS|< 25%.

As reported in Li et al.^63^, NSE and R^2^ exceeded 0.6 and 0.7 for all periods in both calibration and validation, and PBIAS remained within 25% (Table 2). Therefore, SWAT reasonably simulated hydrological processes under climate and LULC changes across the six subzones of the YRB, enabling robust analysis of spatiotemporal drought propagations and mechanisms. The Tangnaiai, Lanzhou, Toudaoguai, Huaxian and Huanyuankou in Table 2 are the hydrological stations in the YRB, and their locations are marked in Fig. 3.

**Table 2:** Results of calibration and validation in three periods measured by the three metrics.

	Calibration			Validation		
	$Ens$	R^2^	PBIAS (%)	$Ens$	R^2^	PBIAS (%)
Baseline						
Tangnaiai	0.76	0.83	3.90	0.83	0.84	5.95
Lanzhou	0.73	0.85	15.71	0.82	0.88	16.93
Toudaoguai	0.68	0.80	12.45	0.74	0.78	11.62
Huaxian	0.60	0.77	− 13.71	0.78	0.85	1.61
Huanyuankou	0.63	0.79	4.88	0.74	0.80	9.70
1990s						
Tangnaiai	0.68	0.75	18.59	0.80	0.91	21.45
Lanzhou	0.74	0.84	14.39	0.84	0.89	13.84
Toudaoguai	0.65	0.74	15.69	0.76	0.77	7.86
Huaxian	0.62	0.71	20.08	0.66	0.70	2.11
Huanyuankou	0.59	0.73	12.43	0.67	0.74	3.87
2000s						
Tangnaiai	0.83	0.83	3.20	0.83	0.86	7.53
Lanzhou	0.78	0.86	8.64	0.76	0.90	8.93
Toudaoguai	0.70	0.76	13.86	0.65	0.76	13.91
Huaxian	0.76	0.76	− 8.64	0.69	0.78	− 7.72
Huanyuankou	0.61	0.72	24.73	0.62	0.71	14.23

### Drought propagation time

**Figure 1:**
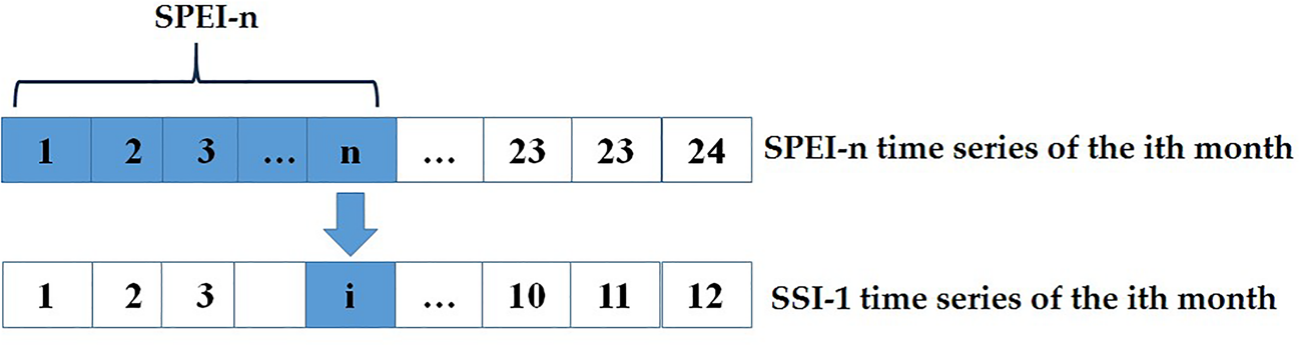
Schematic diagram of propagation time from meteorological drought to hydrological drought.

Meteorological drought indices (e.g. SPEI) are based on precipitation and evapotranspiration, representing deficits in the atmospheric water balance. Hydrological drought indices (e.g. SSI) quantify deficits in surface waters, groundwater, and streamflow that constitute the water sources for human use. As meteorological water deficits propagate through terrestrial systems, it takes time for reduced precipitation and moisture supply to manifest as deficiencies in rivers, lakes, reservoirs and groundwater. Hence, comparing correlation analysis between meteorological and hydrological drought indices provides insight into propagation time from precipitation-evapotranspiration imbalances to resultant surface water, groundwater, and streamflow deficits. This study cross-correlates 1-month Standardized Streamflow Index (SSI-1) time series with 1- to 24-month accumulated Standardized Precipitation Evapotranspiration Index (SPEI) scales using Pearson correlation coefficients. The SPEI accumulation scale exhibiting maximum correlation with SSI-1 is denoted SPEI-n, where n serves as an indicator of drought propagation time (Fig. 1). Longer SPEI accumulation scales strongly correlated with SSI-1 signify extended propagation times, and vice versa. The SPEI-n series for month i is then extracted to represent the meteorological drought conditions n months prior, enabling quantification of propagation lags from meteorological to hydrological drought events.

**Figure 2:**
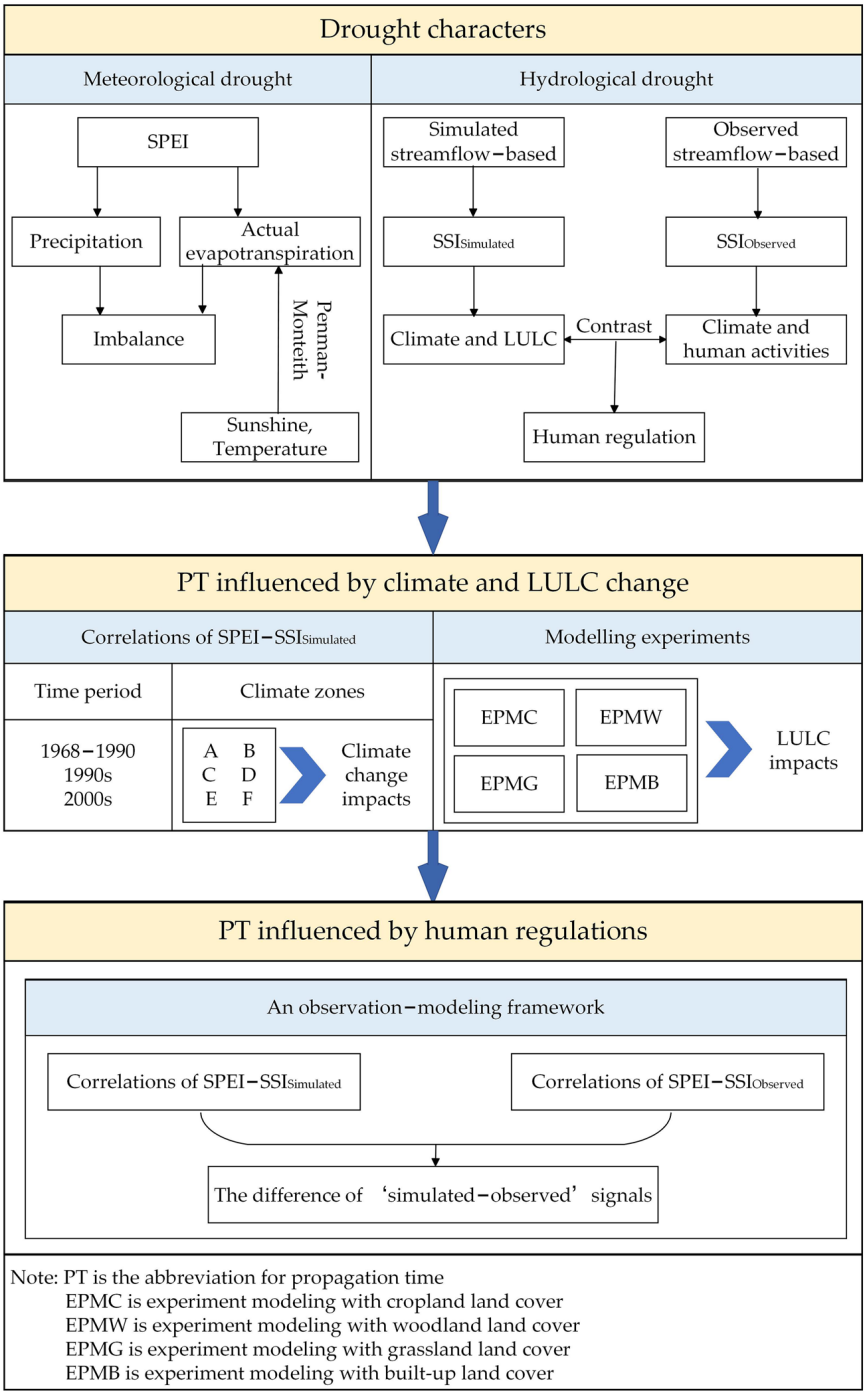
The research flow chart of the study.

To clearly clarify the design ideas in of this study, the research flow chart is provided in Fig. 2.

## Case study and data sources

### Case study of the Yellow River Basin

**Figure 3:**
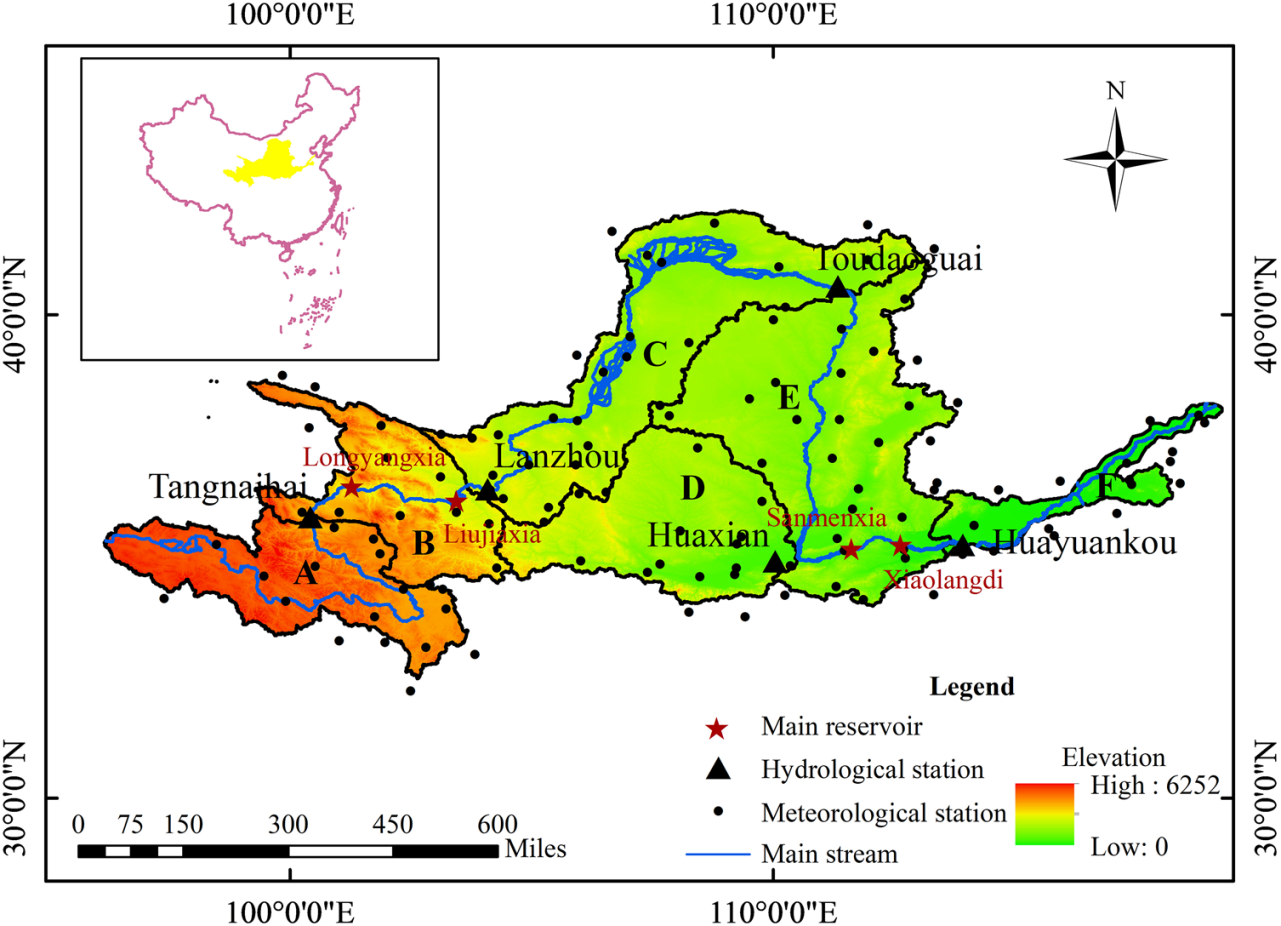
Map depicting the location and topography of the YRB and its six delineated zones. Zone A encompasses a semi-arid to semi-humid region with plateau climatic characteristics. Zone B represents a transitional zone between plateau and mid-temperate climates. Zone C comprises an arid to semi-arid region with mid-temperature climatic features. Zone D constitutes a semi-arid region with warm temperature climate traits. Zone E encompasses a semi-arid to semi-humid zone with temperate continental climate. Zone F constitutes a humid region with temperate monsoonal climate characteristics. The base map of the Chinese map was sourced from the Standard Map Service System of the Ministry of Natural Resources (http://bzdt.ch.mnr.gov.cn/).

The Yellow River Basin (YRB), located between 95°–119°E and 32°–41°N, is the second largest river basin in China and sixth largest globally, with a length of 5464 km and drainage area of 752,443 km^2^ (Fig. 3, generated using ArcMap 10.8 software). Originating on the Tibetan Plateau, the basin meanders eastward through several provinces before discharging into the Bohai Sea.

The YRB has a large area with varied topography, resulting in significant spatial precipitation differences ranging from 1021 mm in the rainy southeast to 123 mm in drier northwest areas^67,68^ Mean annual temperatures vary from − 4 to 14 °C between upper and lower reaches. The YRB renowned for its complex climatic patterns, diverse land use types, and intensive human activities. Hence, the YRB was selected for this study due to its unique environment that allows for the examination of interactions between meteorological and hydrological droughts and the impact of human activities. Water resource allocation and management within the basin are critical for ecological security and socio-economic development in China and the broader East Asian region. Therefore, research on the YRB not only has local significance but also offers insights for global water resource management and drought early warning and defense. To enable analysis of spatial drought dynamics, the basin is delineated into six subzones based on climate characteristics (Fig. 3), with details provided in Li et al.^63^. The hydrological stations such as Tangnaihai, Lanzhou, Toudaoguai, Huaxia and Huayuankou are pivotal for monitoring water flow and quality, providing essential data for flood control, water resource management, and environmental protection. They also serve as key points for dividing the YRB into manageable subzoness for detailed drought analysis.

### Datasets

Daily meteorological data spanning 1966–2010 was acquired from 121 stations across the Yellow River Basin (YRB) (Fig. 3), including precipitation, humidity, wind speed, solar radiation, and maximum/minimum temperatures, obtained from the National Climate Center of China. Monthly naturalized and measured streamflow data for the same period was collected from five hydrological stations (Fig. 3) under the administration of the Yellow River Conservancy Commission. Naturalized streamflow excludes human regulation influences and was used for calibrating SWAT model hydrological parameters. Elevation data was extracted from the 30 m resolution NASA Shuttle Radar Topography Mission (SRTM) Digital Elevation Model (DEM). Soil and land use/land cover (LULC) maps for 1980, 2000, and 2010 were obtained from the Data Center for Resources and Environmental Sciences, Chinese Academy of Sciences.

## Results

### Meteorological drought evolution characteristics

**Figure 4:**
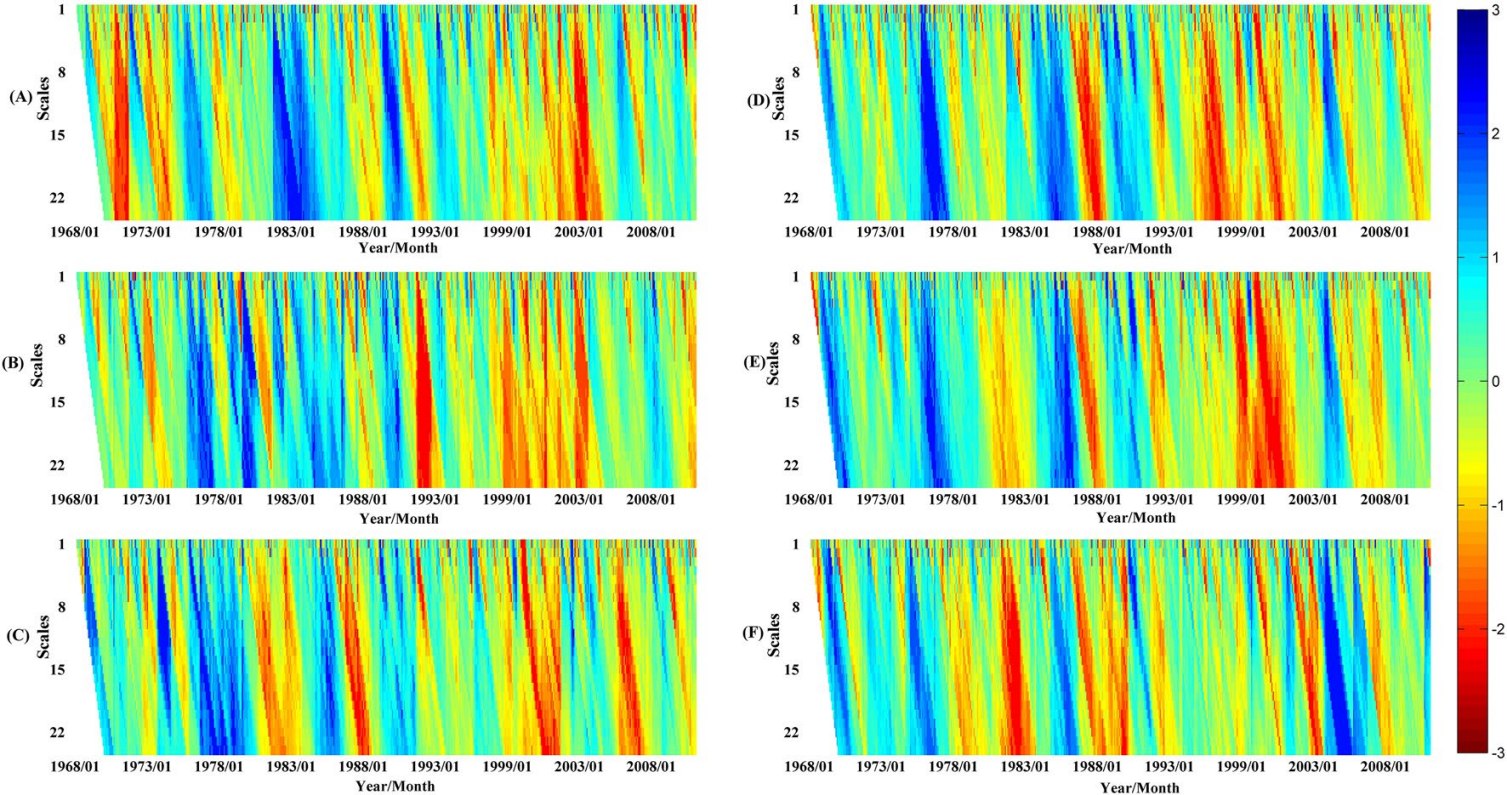
Spatiotemporal distributions of monthly SPEI across YRB subzones at varying accumulation scales, with darker red shading denoting more intense meteorological drought conditions.

Standardized Precipitation Evapotranspiration Index (SPEI) timescales reflect abnormal precipitation and evapotranspiration states over different antecedent accumulation periods. For instance, SPEI-1 and SPEI-3 indicate monthly and seasonal moisture anomalies, respectively. Multi-scale SPEI series (1–24 month accumulations) were thus calculated across YRB subzones from 1968–2010 to examine meteorological drought distribution spatially and temporally. Results are presented in Fig. 4.

Analysis of Fig. 4 reveals increasingly severe meteorological drought across most subzones prior to 2000, especially during the 1990s. However, partial drought relief occurred in the 2000s for many subzones. As an example, subzone D exhibited a shift from extreme 1990s drought to 2000s mitigation. Comparing average annual precipitation and evapotranspiration for the 1968–1990 baseline, 1990s, and 2000s provides insight into drivers. While evapotranspiration showed minimal change (468, 442, 459 mm), precipitation declined sharply from 571 mm (baseline) to 500 mm (1990s), before recovering slightly to 527 mm (2000s). This suggests the 1990s drought primarily resulted from dramatically reduced precipitation, while 2000s easing stemmed from mi-nor precipitation recovery. Figure 4 elucidates spatiotemporal meteorological drought dynamics across the climatically diverse Yellow River Basin, highlighting intense 1990s drought followed by 2000s moisture deficit mitigation for most subzones.

### Hydrological drought evolution characteristics

**Figure 5:**
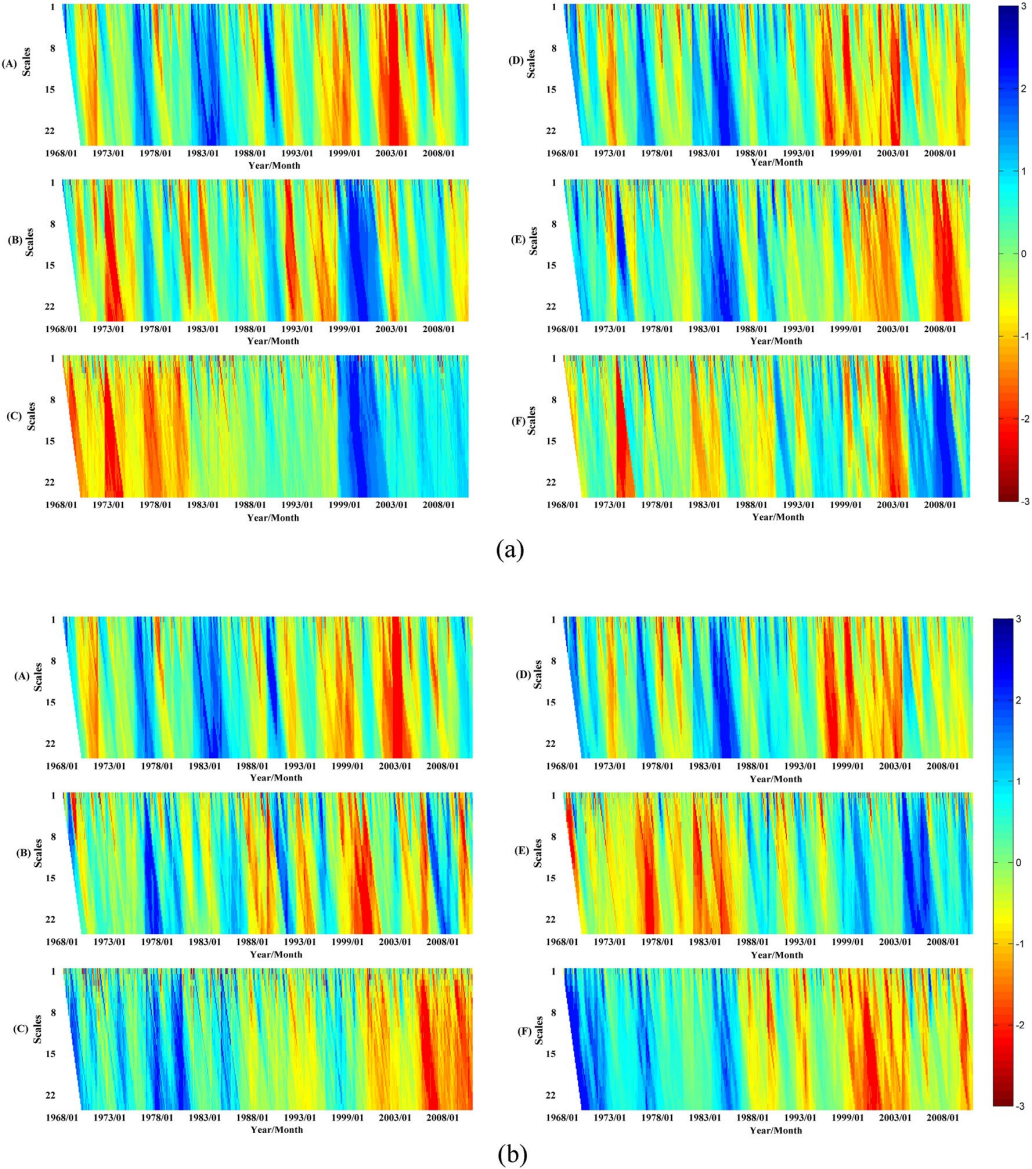
Temporal and spatial evolutions of monthly SSI at different accumulation time-scales under climate-LULC change scenario (**a**), and under climate-human activities scenario (**b**) in the YRB. Darker red color implies more severe hydrological droughts.

Similarly, hydrological drought indexes at different time scales serve as indicators of abnormal conditions in surface water, groundwater, and streamflow over cumulative time periods. Therefore, this study aims to assess the impact of climate and land use/land cover (LULC) changes on hydrological droughts over time by calculating the time series of Standardized Streamflow Index (SSI) with simulated streamflow at various cumulative time-scales (ranging from 1 to 24 months) in six subzones of the YRB from 1968 to 2010. The spatial and temporal evolution of hydrological droughts at different time-scales under the climate-LULC change scenario is presented in Fig. 5a, with darker shades of red indicating more severe drought conditions.

Furthermore, to investigate the influence of human regulations, such as water resources development and utilization, on hydrological droughts, the SSI values with observed streamflow at various cumulative time-scales (1–24 months) in the six sub-zones of the YRB from 1968 to 2010 are also calculated. The spatial and temporal distribution of SSI values at different time-scales under the climate-human activities scenario is illustrated in Fig. 5b, with darker shades of red denoting more severe hydrological droughts.

Under the climate-LULC change scenario (Fig. 5a), the severity of hydrological drought in the cold Plateau region (e.g., subzone A) progressively increased over time. However, in the transition region between the mid-temperate continental and the plateau cold climates (subzone B) and the arid region (subzone C), the hydrological drought conditions improved after 1990. In contrast, since 2000, severe hydrological droughts were observed in the midstream (subzone D and subzone E), while the downstream region (subzone F) experienced some relief after 2005.

When accounting for the impacts of human regulations, such as reservoir storage, irrigation, and industrial and domestic water usage (Fig. 5b), it was observed that, apart from the significant alleviation of hydrological drought in subzone E after 1990, the severity of hydrological drought in the other subzones progressively worsened over time. Particularly since 1990, the occurrence of hydrological drought became more frequent. After 2000, the arid region (subzone C) and the humid region (subzone F) experienced particularly severe hydrological droughts.

### Propagation time influenced by climate variation and LULC change

**Figure 6:**
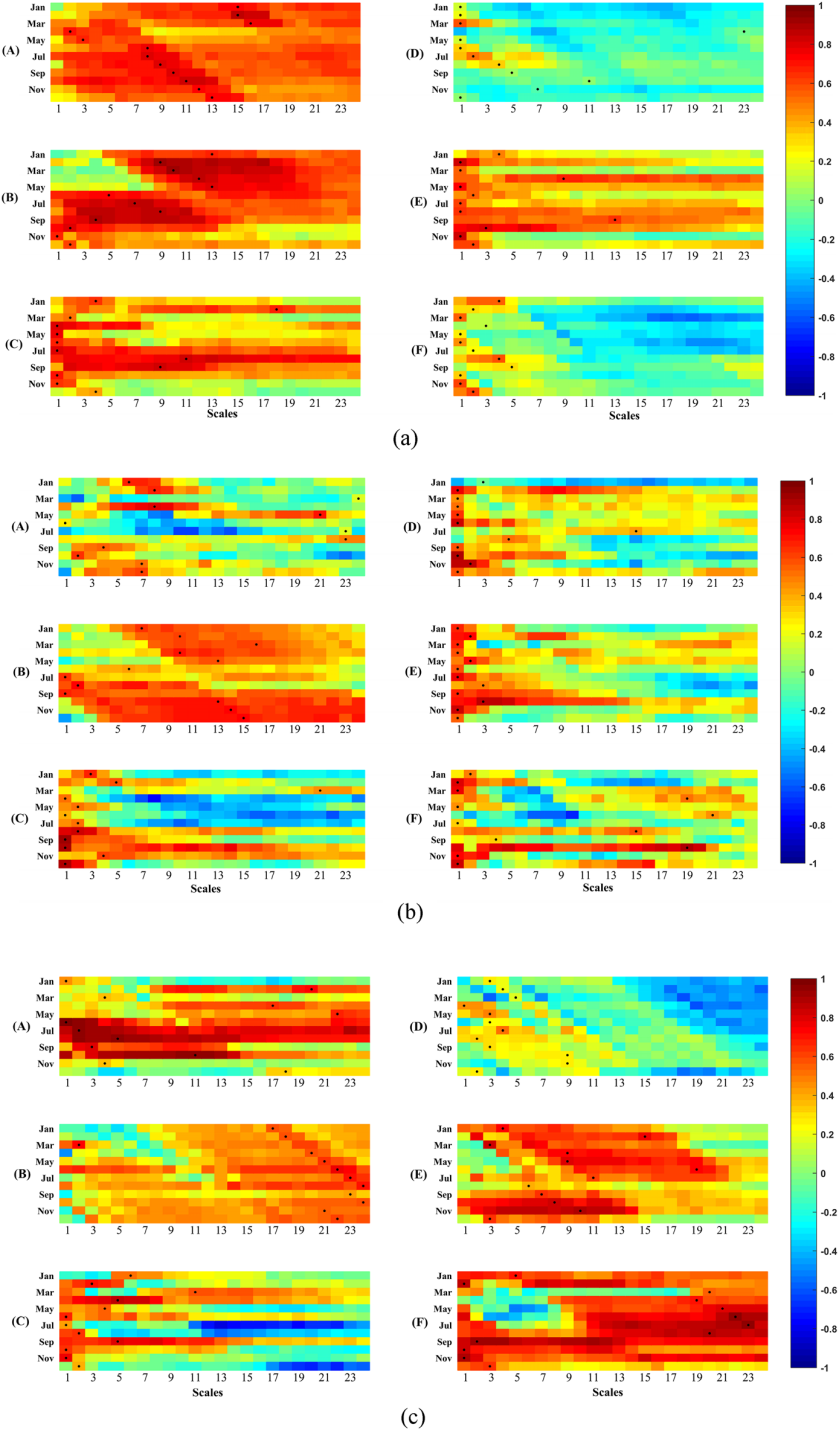
Correlations of SPEI accumulation scales of 1–24 months with SSI-1 under climate-LULC change scenarios over six subzones in the YRB during the baseline period (**a**), the1990s (**b**), and the 2000s (**c**). The black dot denotes the strongest correlation, and the corresponding meteorological cumulative scale is the propagation time.

Figure 6a, b, and c depict heat maps illustrating the correlations between SPEI accumulation scales ranging from 1 to 24 months and SSI-1 under climate-LULC change scenarios across the six subzones within the YRB during the baseline period, the 1990s, and the 2000s. Analyzing the heat maps, it becomes evident that during the baseline period, the correlation between meteorological and hydrological droughts was notably higher in the upstream region compared to the middle and downstream regions. However, in the 1990s and 2000s, this correlation exhibited minimal spatial variability across the subzones. Temporally, the correlation between meteorological and hydrological droughts in the upstream region of the YRB initially declined and then increased. In contrast, the middle and downstream regions displayed a consistent increasing trend. This trend could be attributed to the rapid urban development observed in the middle and downstream regions of the YRB since the 1990s.

Regarding propagation time, noticeable spatial variations were observed within the YRB. Generally, the upstream region exhibited longer propagation times compared to the middle and lower reaches. Temporally, there were negligible differences in propagation time between the baseline period and the 1990s. However, in the 2000s, the propagation time experienced a significant prolongation.

#### Propagation time over time

The propagation time from meteorological to hydrological drought varied substantially over time and space. In the humid subzone F, propagation times fluctuated most over time, providing an example to preliminarily illustrate temporal variations and analyze key influences. During the baseline period, subzone F propagation times were relatively short, concentrated in 1–5 months. Roughly, summer propagation took slightly longer at 4–5 months, likely due to higher summer rainfall frequencies facilitating runoff generation. Compared to the baseline, April, June, August and October propagation times lengthened to 15–21 months in the 1990s. In the 2000s, March to August propagation increased further to 19–23 months.

#### Propagation time in space

Spatially, substantial differences emerged in propagation times, especially summer, between the arid subzone C and humid subzone F in the 2000s. The subzones C and F provide examples to preliminarily illustrate spatial variations in meteorological-to-hydrological drought propagation and discuss potential key influences. Compared to the subzone F, subzone C propagation times were relatively short, concentrated in just 1–6 months.

#### Modeling experiments

**Figure 7:**
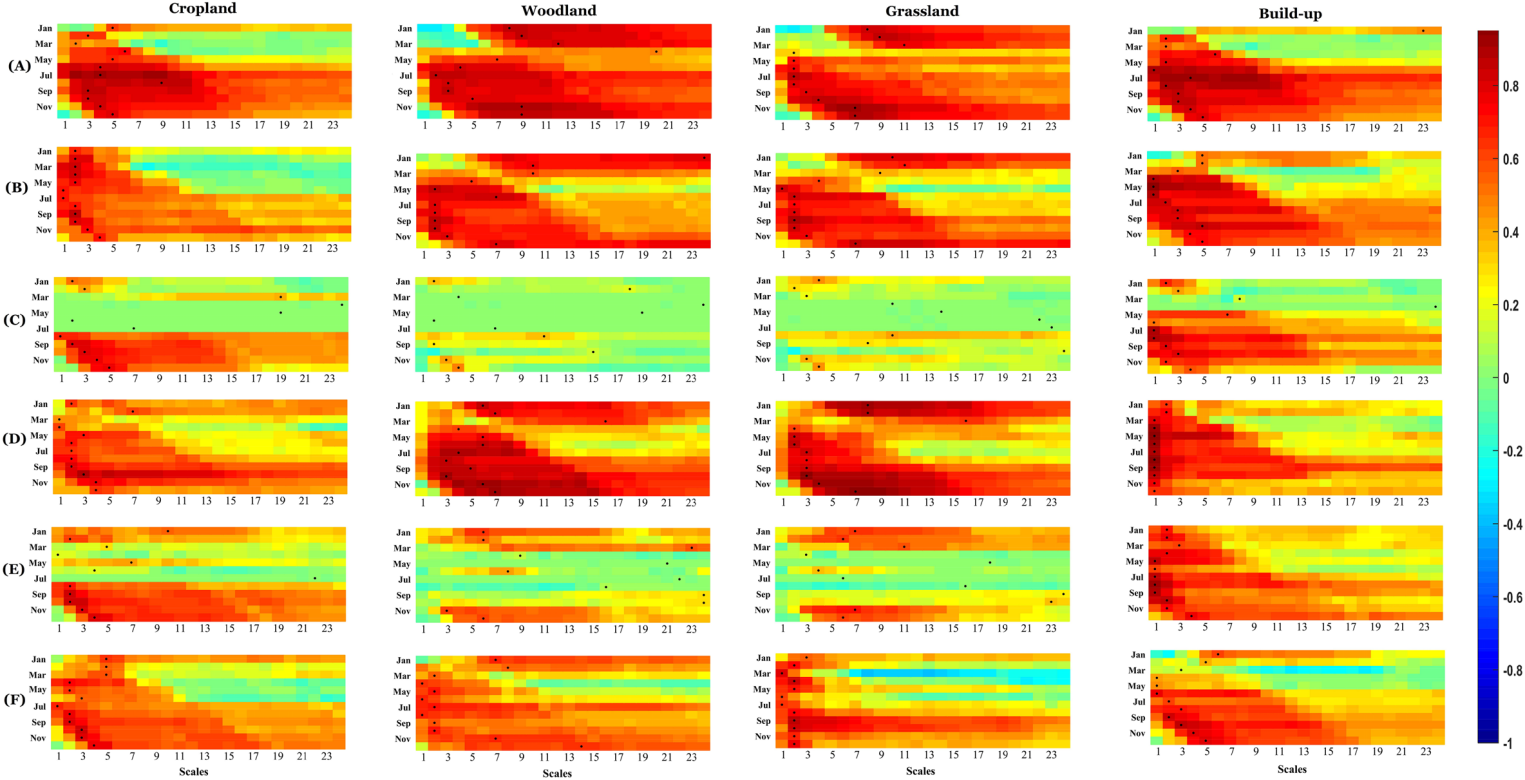
Heat maps of the correlations between SSI-1 and SPEI accumulation scales of 1–24 months across six sub-basins in the YRB under four modeling experiments. The black dot denotes the strongest correlation, representing the corresponding meteorological drought propagation time to hydrological drought.

Based on the preliminary analysis of the propagation mechanism, it is evident that cropland, woodland, grassland and built-up areas have significant influences on the propagation time from meteorological to hydrological drought across the six subzones with differing climate characteristics. In this section, four modeling experiments (EPMC, EPMW, EPMG and EPMB) are conducted using the SWAT model for the latest decade of the impact period (2000s) to examine how these four LULC types affect drought propagation time in the various climate subzones of the YRB. The setup of the four experiments is identical except for differences in the LULC maps representing four extreme LULC scenarios, where all LULC types in the YRB are transformed completely into cropland (EPMC), woodland (EPMW), grassland (EPMG), and built-up areas (EPMB). All other inputs such as DEM and soil data remain constant across the four experiments. Based on the simulated streamflow from these experiments, the SSI at a one month time scale (SSI-1) is calculated for the 2000s across the six YRB sub-zones. The correlations of SPEI-n (n = 1–24 months) with SSI-1 are analyzed for the four modeling experiments as shown in Fig. 7. By comparing the strongest SPEI-n and SSI-1 correlations across the four modeling experiments and six subzones, we can investi-gate how climate and LULC characteristics influence the propagation time from meteorological to hydrological drought in the YRB, thereby further elucidating the physical processes governing the propagation mechanism.

Figure 7 shows high correlations between meteorological and hydrological droughts across the four modeling experiments in the YRB, except for the arid subzone C. Regarding propagation times, noticeable spatial differences emerged among the four experiments, indicating significant impacts of both climate and LULC characteristics. Generally, summer propagation times were shorter across subzones and experiments, suggesting meteorological drought readily propagates to hydrological drought in summer irrespective of climate zone or LULC type. This is likely due to higher summer temperatures increasing evaporation rates and accelerating meteorological-to-hydrological drought propagation.

More broadly, the spatial propagation time variations highlight how local climate and LULC influence meteorological drought propagation into hydrological drought in complex basins like the YRB. The modeling experiments reveal propagation times lengthen under wetter climate regimes and with increased cropland or built-up LULC types. These insights can inform monitoring and management strategies to improve meteorological-to-hydrological drought prediction in different climate and land use contexts.

### Influence of human regulations on propagation time

**Figure 8:**
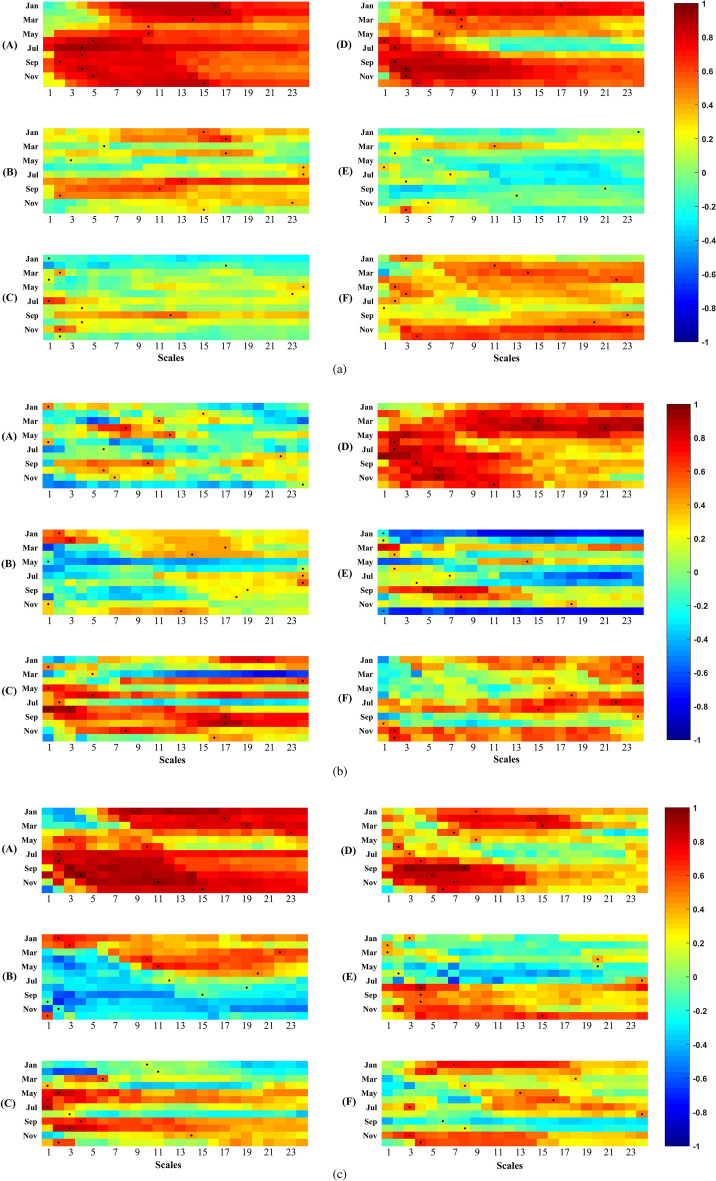
Heat maps of the correlations between SSI-1 and SPEI accumulation scales of 1–24 months under climate-human activity scenarios across six sub-basins in the YRB during the baseline period (**a**), 1990s (**b**), and 2000s (**c**). The black dot denotes the strongest correlation, representing the corresponding meteorological drought propagation time to hydrological drought.

Figure 8a, b, and c display heat maps of the correlations between SSI-1 and SPEI accumulation scales of 1–24 months under climate-human activity scenarios across six sub-basins in the Yellow River Basin during the baseline period, 1990s, and 2000s, respectively. The results show pronounced spatial differences in the correlations between meteorological and hydrological droughts when considering human regulations such as reservoir storage, irrigation, and industrial and domestic water use. Compared to Fig. 6, the correlations decreased substantially in most sub-basins, especially in the middle and lower reaches. The propagation time exhibited an overall prolonging trend but was more dispersed within the year. This suggests that human regulations weaken the link between meteorological and hydrological drought and add complexity to the hydrological processes in the YRB.

Subzone C exhibited relatively greater variability in correlation and propagation time compared to other subzones when considering the influence of human regulations. Therefore, subzone C was analyzed as a case study to determine how human regulations affect the propagation time from meteorological to hydrological drought. In contrast to Figs. 6a–c, 8a–c showed notably decreased correlation, likely due to the water storage provided by two major reservoirs upstream—Longyangxia and Liujiaxia.

## Discussion

### The complex interplay of climate change, land use change, and human regulation on drought propagation mechanism

Temporal changes primarily relate to climate and land use/land cover (LULC) shifts in recent decades. Precipitation and temperature changes in spring and summer, along with LULC transitions quantified using a transition matrix, were analyzed for the subzone F over the past 30 years. The results are shown in Tables 3 and 4, respectively. This analysis reveals how climate variations and LULC changes over recent decades have influenced propagation time lengthening in the humid subzone.

**Table 3:** Average precipitation and temperature in spring and summer in different decades.

Period	Average precipitation (mm)		Average temperature (°C)	
	Spring	Summer	Spring	Summer
Baseline	31.38	126.68	13.75	25.43
1990s	34.68	139.08	14.31	25.92
2000s	34.25	134.94	14.85	25.81

**Table 4:** Transfer percentages of LULC area during of period of 1980–2010.

1980–2010	Cropland (%)	Woodland (%)	Grassland (%)	Water body (%)	Build-up (%)	Bare land (%)
Cropland	81.26	2.04	2.67	2.08	11.71	0.25
Woodland	17.87	66.35	12.91	0.88	1.91	0.08
Grassland	31.32	14.84	47.68	2.52	2.96	0.67
Water body	50.00	2.73	3.95	38.41	3.96	0.98
Build-up	26.62	1.08	1.42	2.08	68.66	0.15
Bare land	58.86	4.07	5.58	6.13	8.84	18.50

Table 3 shows little change in average spring and summer precipitation and temperature over time in the subzone F, indicating climate change may not be the primary driver prolonging propagation times in these seasons. The LULC transition matrix in Table 4 reveals that from 1980 to 2010, substantial woodland and grassland area trans-formed into cropland, at 17.81% and 31.32% respectively. Meanwhile, 11.71% of cropland converted to built-up area. These transitions likely accelerated surface runoff generation in the hydrological cycle, as croplands and built-up areas have lower surface resistance and faster flow velocities compared to woodlands and grasslands when it rains. Consequently, the LULC changes appear to have prolonged meteorological-to-hydrological drought propagation times in the humid subzone, especially during rainy spring and summer seasons.

**Figure 9:**
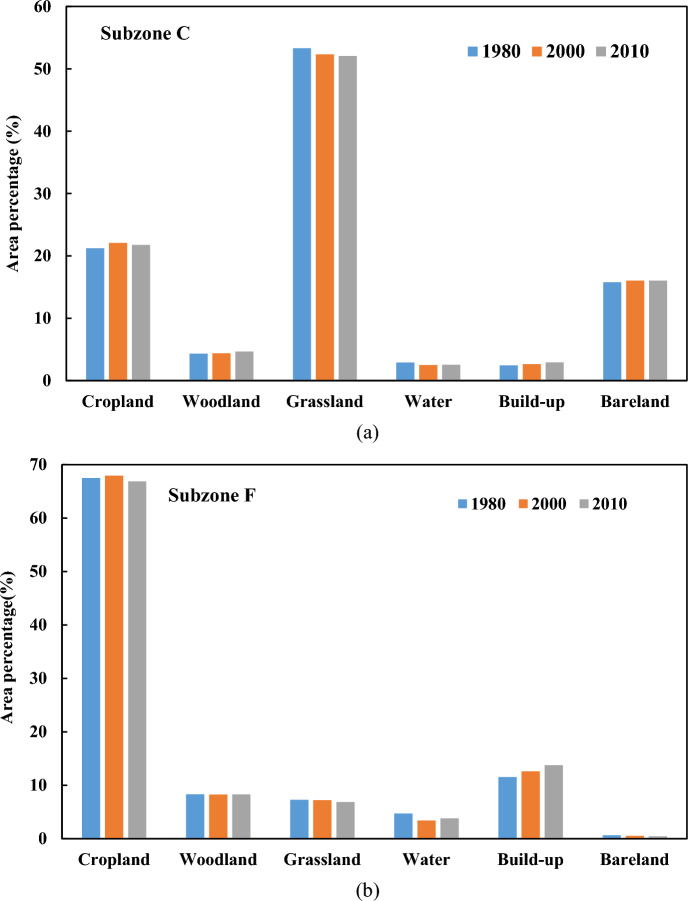
Area percentages of LULC types in subzone C (**a**) and subzone F (**b**).

This discrepancy likely relates to contrasting climate and land surface characteristics. The arid subzone C in the Loess Plateau receives under 300 mm annual precipitation, while the humid subzone F exceeds 600 mm. Additionally, the subzone C is dominated by grasslands, whereas croplands prevail in the subzone F (Fig. 9a and b). Croplands clearly generate surface runoff more readily following frequent rainstorms compared to grasslands in humid regions. Consequently, different climate regimes and land use likely drive longer meteorological-to-hydrological drought propagation times in the humid cropland versus the arid grassland.

More interestingly, substantial propagation time differences emerged across climate zones despite similar underlying land surfaces (LULC types), especially between arid and humid regions. For instance, propagation times in the arid subzone C were shorter in the cropland (EPMC) and built-up (EPMB) experiments versus the woodland (EPMW) and grassland (EPMG) experiments. Conversely, the humid subzone F showed longer propagation times under cropland and built-up versus woodland and grassland experiments.

These differences likely stem from how underlying surfaces affect surface water and groundwater dynamics differently in arid versus humid climates. The EPMC, EPMB, EPMW and EPMG experiments reflect cropland, built-up, woodland and grassland land covers, respectively. Clearly, croplands and built-up areas generate more surface runoff when it rains compared to woodlands and grasslands. This runoff can temporarily delay hydrological drought onset in rainy humid regions like the subzone F, effectively prolonging meteorological-to-hydrological drought propagation. This aligns with earlier results showing lengthened propagation times when wood-lands/grasslands converted to croplands in the subzone F.

In contrast, woodlands and grasslands better retain soil water and recharge groundwater versus croplands and built-up areas. This alleviates hydrological drought in drier regions with less rainfall. Consequently, woodlands and grasslands likely cause slower precipitation deficit propagation into hydrological drought in arid regions like the subzone C compared to croplands and built-up areas.

Reservoir operations can smooth hydrographs, resulting in higher low flows and lower peak flows, thus disturbing the natural rainfall-runoff process. Moreover, propagation time, especially in the 2000s, was prolonged under human regulation, primarily attributable to the effective regulation of reservoirs often designed to mitigate water deficits, thereby prolonging the meteorological to hydrological drought propagation time to some degree.

Overall, this research underscores the intricate relationship between climate change, land use change, and human regulation in influencing drought dynamics. It reveals that while climate change sets the broader stage for drought occurrence, land use practices and human regulations, such as reservoir management, play pivotal roles in modulating drought propagation times across different climatic zones. In humid regions, urbanization and agricultural expansion can increase surface runoff, delaying the onset of hydrological droughts and extending drought cycles. Conversely, in arid zones, natural vegetation like forests and grasslands are crucial in maintaining soil moisture and supporting groundwater recharge, thus mitigating hydrological droughts and decelerating the transition from meteorological to hydrological drought. The operation of reservoirs introduces another layer of complexity, as it can disrupt natural hydrological patterns, sometimes prolonging droughts by altering rainfall-runoff processes. This multifaceted impact is most evident in the way land use changes and human regulatory measures, such as the strategic operation of reservoirs, have been found to significantly influence the temporal and spatial characteristics of drought propagation, highlighting the need for integrated management approaches that consider the diverse effects of these factors.

### Uncertainty estimation the SWAT model

While comparing simulated and observed streamflow enables evaluating SWAT model performance, uncertainties can arise from limitations in capturing complex hydrological processes^69^. For example, SWAT relies on calibrated parameters to represent basin-specific water budgets, introducing subjectivity and constraints from observational data availability in parameter selection and validation. Additionally, conceptual model structural uncertainty stems from the simplification of intricate hydrological process representations^70^. Input data uncertainty can also emerge from errors in measurement, resolution, or spatial interpolation^9,71^.

To reduce such uncertainties in future work, several approaches could improve scientific understanding of the intricate YRB water cycle dynamics. Implementation of flexible Bayesian frameworks that allow model parameters and process representations to vary spatially and evolve temporally, based on assimilation of integrated meteorological, ecological, agricultural, and hydrological datasets, may overcome structural deficiencies. Tight coupling of subsurface and groundwater compartments could enhance representation of base-flow and water table dynamics governing hydrological memory and drought persistence. Additionally, dynamic model parameter estimation methods and fusion with complementary models can help constrain uncertainties.

### Application and limitation of the research results

The findings in this study can advance understanding of how climate types, land surface characteristics, and human regulations like water resource development influence drought evolution and propagation across complex basins such as the YRB. Such insights have policy relevance for land use planning and water management strategies aimed at facilitating effective drought prediction and mitigation. The results highlight the utility of considering local climate, land cover, and human factors when developing integrated approaches to monitor and manage droughts in complex basins like the YRB.

However, it is important to acknowledge key limitations of this study. Namely, the current analysis lack of considering potential nonlinearities in drought propagation processes. In reality, the propagation from meteorological to hydrological drought involves nonlinear propagation characteristics in addition to linear connections. Hence, a promising avenue for future work is to elucidate nonlinear propagation mechanisms linking different drought types under changing climate and human pressures. Analyses quantifying nonlinear drought impacts and feedbacks could provide deeper scientific understanding to advance predictive capabilities and inform adaptive water management strategies. Coupling empirical analyses with process-based models may aid in disentangling climate change and human controls on coupled meteorological–hydrological drought dynamics across complex basins like the YRB.

## Conclusion

Investigating linkages between meteorological and hydrological drought events and diagnosing drivers governing their propagation lay groundwork for developing integrated forecasting frameworks leveraging meteorological warnings for predicting emergent hydrological extremes. This study uses an observation-modeling framework integrating the Standardized Precipitation Evapotranspiration Index (SPEI) and Standardized Streamflow Index (SSI) under observed and simulated scenarios to quantify meteorological-to-hydrological drought connections and isolate propagation mechanisms. With the intensely-managed YRB as a testbed, key conclusions regarding spatiotemporal drought dynamics are as follows:

1. Meteorological drought severity rose from 1968 to 2000 before stabilizing post-2000, apart from sub-humid zones where agricultural expansion has maintained atmospheric moisture losses. Hydrological drought trends showed higher climate dependency, with alleviation upstream but continued intensification downstream.
2. Summer propagation times from meteorological to hydrological drought events are shorter than other seasons across climate zones, attributed to heightened evaporation rates accelerating atmospheric moisture deficit transfer.
3. Longer propagation time arise in humid croplands and built-up regions relative to humid grasslands/woodlands because the former's surface runoff hinders onset of rainy season hydrological droughts. Arid croplands and built-up areas contrastingly have shorter meteorological–hydrological drought propagation time than arid grasslands/woodlands due to the latter's soil water retention slightly buffering hydrological drought.
4. Human regulations via extensive water storage infrastructure prolong basin-wide propagation times, especially post-2000, as controlled reservoir releases overcome natural water limitations and delay meteorological drought realization as hydrological droughts.

## Data Availability

The datasets generated and /or analyzed during the current study are not publicly available due to privacy but are available from the corresponding author on reasonable request.

## References

[1] Sheffield J, Wood EF (2011). Drought: Past Problems and Future Scenarios.

[2] Van Loon AF, Laaha G (2015). Hydrological drought severity explained by climate and catchment characteristics. J. Hydrol..

[3] Barker LJ, Hannaford J, Chiverton A, S C (2016). From meteorological to hydrological drought using standardised in-dicators. Hydrol. Earth Syst. Sci. Discuss..

[4] Li, J. *et al*. Robust meteorological drought prediction using antecedent SST fluctuations and machine learning. *Water Resour Res*. **57** (2021).

[5] Wilhite DA, Glantz MH (1985). Understanding: The drought phenomenon: The role of definitions. Water Int..

[6] Yu M, Cho Y, Kim TW, Chae HS (2018). Analysis of drought propagation using hydrometeorological data: From me-teorological drought to agricultural drought. J. Korea Water Resour. Assoc..

[7] Wu J, Miao C, Lei CX, Li H (2018). Meteorological and hydrological drought on the Loess Plateau, China: Evolutionary characteristics, impact, and propagation. J. Geophys. Res. Atmos..

[8] Abro MI (2022). Esti-mation of a trend of meteorological and hydrological drought over Qinhuai River Basin. Theor. Appl. Climatol..

[9] Huang S, Li P, Huang Q, Leng G, Hou B, Ma L (2017). The propagation from meteorological to hydrological drought and its potential influence factors. J. Hydrol..

[10] Li YF (2022). High-resolution propagation time from meteorological to agricultural drought at multiple levels and spatiotemporal scales. Agric. Water Manag..

[11] Wang M (2021). Separating the effects of climate change and human activities on drought propagation via a natural and human-impacted catchment comparison method. J. Hydrol..

[12] He Y, Qiu H, Song J, Zhao Y, Zhang L, Hu S, Hu Y (2019). Quantitative contribution of climate change and human activities to runoff changes in the Bahe River watershed of the Qinling Mountains, China. Sustain. Cities Soc..

[13] Zhang T, Su X, Wu L (2023). Integrating multiple comparison methods for attributing hydrological drought evolution and drought propagation: The impact of climate change cannot be ignored. J. Hydrol..

[14] Ding, Y. B., Xu, J. T., Wang, X. W., Cai, H. J., Zhou, Z. Q., Sun, Y. N. & Shi, H. Y. Propagation of meteorological to hydrological drought for different climate regions in China. *J. Environ. Manag*. **283** (2021). 10.1016/j.jenvman.2021.111980 33477095

[15] Meng, D. *et al*. Propagation characteristics and mechanism from meteorological to agricultural drought in various seasons. *J. Hydrol*. **610** (2022).

[16] Ma F, Luo L, Ye A, Duan Q (2019). Drought characteristics and propagation in the semiarid Heihe River Basin in Northwestern China. J. Hydrometeorol..

[17] Li Y, Chang J, Luo L, Wang Y, Guo A, Ma F, Fan J (2019). Spatiotemporal impacts of land use land cover changes on hydrology from the mechanism perspective using SWAT model with time-varying parameters. Hydrol. Res..

[18] Taiwo BE (2023). Monitoring and predicting the influences of land use/land cover change on cropland characteristics and drought severity using remote sensing techniques. Environ. Sustain. Indic..

[19] Wu J, Chen X, Yao H, Gao L, Chen Y, Liu M (2017). Non-linear relationship of hydrological drought responding to meteorological drought and impact of a large reservoir. J. Hydrol..

[20] Wu JF, Yuan X, Yao HX, Chen XH, Wang GX (2021). Reservoirs regulate the relationship between hydrological drought recovery water and drought characteristics. J. Hydrol..

[21] Chenzhong, L. & Jie, T. U. Construction of water conservancy is to study the impact of agricultural flood and drought disaster area: A case study of reservoir construction. *Ecol. Econ*. (2012).

[22] Cai S (2021). Spatiotemporal characteristics of agricultural droughts based on soil moisture data in Inner Mongolia from 1981 to 2019. J. Hydrol..

[23] Zhou, Z., Shi, H., Fu, Q., Ding, y., Li, T., Wang, Y. & Liu, S. Characteristics of propagation from meteorological drought to hydrological drought in the Pearl River Basin. *J. Geophys. Res. Atmos.* **126** (2021) 10.1016/j.scitotenv.2023.165618 37474042

[24] Hu CH, Zhao LX, Wang YX, Xue X, Wu L (2016). Analysis of the relationship between the meteorological, agriculture and hydrological drought. Meteorol. Environ. Sci..

[25] Li Y, Huang Y, Li Y, Zhang H, Deng Q, Fan J, Wang X (2023). Temporal and spatial propagation characteristics of the meteorological, agricultural and hydrological drought system in different climatic conditions within the framework of the watershed water cycle. Water.

[26] Chang J, Li Y, Yuan M, Wang Y (2017). Efficiency evaluation of hydropower station operation: A case study of long-Yangxia station in the yellow river, China. Energy.

[27] Wagener, T., Sivapalan, M., Troch, P. A., McGlynn, B. L., Harman, C. J., Gupta, H. V. & Wilson, J. S. The future of hydrology: An evolving science for a changing world. *Water Resour. Res.* **46** (2010).

[28] Van Loon AF, Rangecroft S, Coxon G, Breña Naranjo JA, Van Ogtrop F, Van Lanen HA (2019). Using paired catchments to quantify the human influence on hydrological droughts. Hydrol. Earth Syst. Sci..

[29] Van Loon AF, Van Lanen HA (2013). Making the distinction between water scarcity and drought using an observation-modeling framework. Water Resour. Res..

[30] Rangecroft S, Van Loon AF, Maureira H, Verbist K, Hannah DM (2019). An observation-based method to quantify the human influence on hydrological drought: Upstream–downstream comparison. Hydrol. Sci. J..

[31] Liu, Y., Ren, L., Zhu, Y., Yang, X., Yuan, F., Jiang, S. & Ma, M. Evolution of hydrological drought in human disturbed areas: A case study in the Laohahe Catchment, northern China. *Adv. Meteorol*. **2016** (2016).

[32] Li Q, Zhou J, Zou W, Zhao X, Huang P, Wang L, Zhu G (2021). A tributary-comparison/method to quantify the human influence on hydrological drought. J. Hydrol..

[33] Omer A, Zhuguo M, Zheng Z, Saleem F (2020). Natural and anthropogenic influences on the recent droughts in Yellow River Basin, China. Sci. Total Environ..

[34] Wang F, Wang Z, Yang H, Zhao Y (2018). Study of the temporal and spatial patterns of drought in the Yellow River basin based on SPEI. Sci. China Earth Sci..

[35] Zhang, Q., Miao, C., Guo, X., Gou, J. & Su, T. Human activities impact the propagation from meteorological to hydrological drought in the Yellow River Basin, China. *J. Hydrol*. 129752 (2023).

[36] Adeaga O (2013). Drought risks and impact on water resources in part of northern Nigeria. Clim. Land Surf. Chang. Hydrol. Proc. H..

[37] Jiao, Y., Yuan, X. & Yang, D. Changes in the characteristics of hydrological droughts over a semi-arid watershed within Yellow River basin. EGU General Assembly Conference Abstracts. 15687 (2017).

[38] Ma M, Ren L, Singh VP, Fei Y, Lu C, Yang X (2016). Hydrologic model-based palmer indices for drought character-ization in the Yellow River Basin, China. Stoch. Environ. Res. Risk Assess..

[39] Li, B. Q., Zhu, C. C., Liang, Z. M., Wang, G. Q. & Zhang, Y. Connections between meteorological and hydrological droughts in a semi-arid basin of the middle yellow river. In *8th International Water Resources Management Conference of ICWRS*. **379**, 403–407 (2018).

[40] Zhu Y, Chang J, Huang S, Huang Q (2016). Characteristics of integrated droughts based on a nonparametric standardized drought index in the yellow river basin, China. Hydrol. Res..

[41] Wu D, Yan DH, Yang GY, Wang XG, Xiao WH, Zhang HT (2013). Assessment on agricultural drought vulnerability in the yellow river basin based on a fuzzy clustering iterative model. Nat. Hazards.

[42] Geng G, Yang R, Liu L (2022). Downscaled solar-induced chlorophyll fluorescence has great potential for monitoring the response of vegetation to drought in the Yellow River Basin, China: Insights from an extreme event. Ecol. Indic..

[43] Li Z, Wang Y, Zhang H, Chang J, Yu Y (2022). Runoff response to changing environment in Loess Plateau, China: Implications of the influence of climate, land use/land cover, and water withdrawal changes. J. Hydrol..

[44] Stagge JH, Kohn I, Tallaksen LM, Stahl K (2015). Modeling drought impact occurrence based on meteorological drought indices in Europe. J. Hydrol..

[45] Chang J, Li Y, Wang Y, Yuan M (2016). Copula-based drought risk assessment combined with an integrated index in the Wei river basin, China. J. Hydrol..

[46] Wang H, He B, Zhang Y, Huang L, Chen Z, Liu J (2018). Response of ecosystem productivity to dry/wet conditions indicated by different drought indices. Sci. Total Environ..

[47] Khan MI, Dong L, Qiang F, Faiz MA (2018). Detecting the persistence of drying trends under changing climate conditions using four meteorological drought indices. Meteorol. Appl..

[48] Chen H, Sun J (2015). Changes in drought characteristics over china using the standardized precipitation evapotranspiration index. J. Clim..

[49] Zarch MAA, Sivakumar B, Sharma A (2015). Droughts in a warming climate: A global assessment of standardized precipitation index SPI) and reconnaissance drought index (SPEI). J. Hydrol..

[50] Shang JD, Zhao B, Hua HB, Wei JR, Qin GY, Chen GJ (2023). Application of informer model based on SPEI for drought forecasting. Atmosphere.

[51] Lang D, Zheng J, Shi J, Liao F, Ma X, Wang W, Zhang M (2017). A comparative study of potential evapotranspiration estimation by eight methods with FAO Penman-Monteith method in southwestern China. Water.

[52] De Bruin HAR (1983). A model for the Priestley-Taylor parameter α. J. Appl. Meteorol. Climatol..

[53] Hargreaves GH, Allen RG (2003). History and evaluation of Hargreaves evapotranspiration equation. J. Irrig. Drain. Eng..

[54] Hoekema DJ, Sridhar V (2011). Relating climatic attributes and water resources allocation: A study using surface water supply and soil moisture indices in the snake river basin, idaho. Water Resour. Res..

[55] Palmer, W. C. Meteorological drought, Res. Pap. 45, 58, U.S. Weather Bur (1965).

[56] Wang DB, Hejazi M, Cai XM, Valocchi AJ (2011). Climate change impact on meteorological, agricultural, and hydrological drought in central Illinois. Water Resour. Res..

[57] Svensson C, Hannaford J, Prosdocimi I (2017). Statistical distributions for monthly aggregations of precipitation and streamflow in drought indicator applications. Water Resour. Res..

[58] Arnold JG, Srinivasan R, Muttiah RS, Williams JR (1998). Large area hydrologic modeling and assessment part i: Model development. J. Am. Water Resour. Assoc..

[59] Zhang J, Li L, Li D, Deng W (2015). Summer droughts in the northern yellow river basin in association with recent arctic ice loss. Int. J. Climatol..

[60] Li YY, Chang JX, Wang YM, Jin WT, Guo AJ (2016). Spatiotemporal impacts of climate, land cover change and direct human activities on runoff variations in the Wei river basin, China. Water.

[61] Woldesenbet TA, Elagib NA, Ribbe L, Heinrich J (2017). Hydrological responses to land use/cover changes in the source region of the upper blue nile basin, Ethiopia. Sci. Total Environ..

[62] Lin F, Chen XW, Yao HX, Lin FY (2022). SWAT model-based quantification of the impact of land-use change on forest-regulated water flow. Catena.

[63] Li YY (2020). Hydrological drought evolution with a nonlinear joint index in regions with significant changes in underlying surface. J. Hydrol..

[64] Nash JE, Sutcliffe JV (1970). River flow forecasting through conceptual models part I—A discussion of principles. J. Hydrol..

[65] Gupta HV, Sorooshian S, Yapo PO (1999). Status of automatic calibration for hydrologic models: Comparison with multilevel expert calibration. J. Hydrol. Eng..

[66] Moriasi DN, Arnold JG, Liew MWV, Bingner RL, Harmel RD, Veith TL (2007). Model evaluation guidelines for systematic quantification of accuracy in watershed simulations. Trans. Asabe.

[67] Zhang Q, Xu CY, Chen YD, Ren LL (2011). Comparison of evapotranspiration variations between the Yellow River and Pearl River basin, China. Stoch. Environ. Res. Risk Assess..

[68] She D, Xia J, Song J, Hong D, Chen J, Long W (2013). Spatio-temporal variation and statistical characteristic of extreme dry spell in yellow river basin, China. Theor. Appl. Climatol..

[69] Jehanzaib M, Shah SA, Kim JE, Kim TW (2023). Exploring spatio-temporal variation of drought characteristics and propagation under climate change using multi-model ensemble projections. Nat. Hazards.

[70] Shah SA, Jehanzaib M, Park KW, Choi S, Kim TW (2023). Evaluation and decomposition of factors responsible for alteration in streamflow in lower watersheds of the han river basin using different Budyko-based functions. KSCE J. Civ. Eng..

[71] Barker LJ, Hannaford J, Chiverton A, Svensson C (2016). From meteorological to hydrological drought using standardised indicators. Hydrol. Earth Syst. Sci..

